# Identification of the Causative Gene for Simmental Arachnomelia Syndrome Using a Network-Based Disease Gene Prioritization Approach

**DOI:** 10.1371/journal.pone.0064468

**Published:** 2013-05-16

**Authors:** Shihui Jiao, Qin Chu, Yachun Wang, Zhenquan Xie, Shiyu Hou, Airong Liu, Hongjun Wu, Lin Liu, Fanjun Geng, Congyong Wang, Chunhua Qin, Rui Tan, Xixia Huang, Shixin Tan, Meng Wu, Xianzhou Xu, Xuan Liu, Ying Yu, Yuan Zhang

**Affiliations:** 1 Key Laboratory of Agricultural Animal and Breeding, National Engineering Laboratory for Animal Breeding, College of Animal Science and Technology, China Agricultural University, Beijing, China; 2 Institute of Animal Husbandry and Veterinary Medicine, Beijing Academy of Agriculture and Forestry Sciences, Beijing, China; 3 Anshan Hengli Dairy Farm, Anshan, Liaoning, China; 4 Xiertala Breeding Farm, Hailaer Farm Buro, Hailaer, Inner Mongolia, China; 5 Hailaer Farm Buro, Hailaer, Inner Mongolia, China; 6 Beijing Dairy Cattle Centre, Beijing, China; 7 Dingyuan Seedstock Bulls Breeding Ltd. Company, Zhengzhou, Henan, China; 8 Ningxia Sygen BioEngineering Research Center, Yinchuan, Ningxia, China; 9 Xinjiang General Livestock Service, Urumqi, Xinjiang, China; 10 College of Animal Science, Xinjiang Agriculture University, Urumqi, Xinjiang, China; 11 Xinjiang Tianshan Animal Husbandry Bio-engineering Co. Ltd, Urumqi, Xinjiang, China; 12 Dalian Xuelong Industry Limited Group, Dalian, Liaoning, China; University of Sydney, United States of America

## Abstract

Arachnomelia syndrome (AS), mainly found in Brown Swiss and Simmental cattle, is a congenital lethal genetic malformation of the skeletal system. In this study, a network-based disease gene prioritization approach was implemented to rank genes in the previously reported ∼7 Mb region on chromosome 23 associated with AS in Simmental cattle. The top 6 ranked candidate genes were sequenced in four German Simmental bulls, one known AS-carrier ROMEL and a pooled sample of three known non-carriers (BOSSAG, RIFURT and HIRMER). Two suspicious mutations located in coding regions, a mis-sense mutation c.1303G>A in the bystin-like (*BYSL*) gene and a 2-bp deletion mutation c.1224_1225delCA in the molybdenum cofactor synthesis step 1 (*MOCS1*) gene were detected. Bioinformatic analysis revealed that the mutation in *MOCS1* was more likely to be the causative mutation. Screening the c.1224_1225delCA site in 383 individuals from 12 cattle breeds/lines, we found that only the bull ROMEL and his 12 confirmed progeny carried the mutation. Thus, our results confirm the conclusion of Buitkamp et al. that the 2-bp deletion mutation c.1224_1225delCA in exon 11 of the *MOCS1* gene is causative for AS in Simmental cattle. Furthermore, a polymerase chain reaction-restriction fragment length polymorphism (PCR-RFLP) was developed to detect the causative mutation.

## Introduction

Arachnomelia syndrome (AS, OMIA Phene ID 139, Group 000059), inherited as a monogenic autosomal recessive trait, is a lethal congenital abnormality of the skeletal system in cattle. Affected calves are usually stillborn and characterized by complex anomalies including facial deformities, skeletal malformation of the vertebral column, and abnormally thin and prolonged legs, giving a spidery appearance [1]–[7]. The sporadic occurrence of AS was first reported by Rieck and Schade [1] in the Simmental, Holstein-Friesian and German Red and White cattle populations in 1975. Arachnomelia was reported in Brown Swiss in 1987 [2] and again in Simmental in 2005 [3]. Extensive use of artificial insemination created the opportunity for the mutation to spread more rapidly, drawing the attention of breeders and researchers.

In Brown Swiss, AS was initially mapped to bovine chromosome (BTA) 5 [2], and the causal mutation was a single base insertion c.363–364insG in the fourth exon of the sulfite oxidase (*SUOX*) gene, which led to a frame-shift and a predicted premature stop codon [4]. However, research conducted on AS in Simmental cattle indicated that the causal mutation may be located in a region between microsatellite markers *DIK4340* and *BM1815* on BTA 23, covering a length of about 7 Mb [5]. In 2011, by using a dense set of microsatellite markers, the region was further refined, and comparative sequencing of the genes in the region revealed that a 2-bp deletion in the bovine molybdenum cofactor synthesis step 1 (*MOCS1*) gene was the functional mutation responsible for AS in Simmental cattle [6]. Since 2005, China has imported semen from top ranking German Simmental bulls to improve milk and meat production in Chinese cattle population. Semen of the famous German Simmental bull ROMEL, a carrier of AS [3], [5], was also introduced into China in 2006. To prevent the spread of this lethal genetic disorder among the Chinese cattle, it is crucial to establish a method to identify AS-carriers.

Candidate gene prioritization by data integration is a valid method to rank putative genes according to their relevance to the biological process of interest, which could help select the most promising candidate or greatly reduce the number of genes to be considered in follow-up studies [8]–[10]. In this study, this method was used to search for the causal mutation of AS in Simmental as an alternative procedure of the fine mapping approach adopted by Buitkamp et al. [6]. All protein coding genes located in the previously reported ∼7 Mb region were ranked. Top ranked candidate genes were sequenced and analyzed to investigate mutations underlying AS, and consequently, a method for detecting carriers of AS in the Simmental breed was established.

## Results

### Ranking of candidate genes

According to NCBI Map Viewer (http://www.ncbi.nlm.nih.gov/mapview/), there were a total of 83 genes located in the ∼7 Mb mapped interval on BTA 23 reported by Buitkamp et al. [5]; however, only 27 genes could be prioritized by the phenotypic profile. The relative importance of the 27 positional candidates was ranked by the relevance score (z-score), which represented an overall enrichment signal for the association of the candidate gene to AS (Table S1). Genes with a large positive z-score are strongly associated with AS.

Genes were evaluated in ranking order based on the z-score. Six highly prioritized genes were sequenced, namely bystin-like (*BYSL*), TATA box binding protein (TBP)-associated factor (*TAF8*), ring finger protein 8 (*RNF8*), cyclin-dependent kinase inhibitor 1A (*CDKN1A*), TBC1 domain family, member 22B (*TBC1D22B*) and *MOCS1*. Since a potential causative mutation was discovered in *MOCS1*, genes ranking lower than *MOCS1* were not evaluated.

### 2-bp deletion in MOCS1 gene may be responsible for AS

All exons and flanking intronic regions of the six genes were amplified, sequenced and aligned with reference sequences to explore mutations. Total length of the fragments sequenced was about 30.8 kb and 48 mutations were detected. Out of the 48 mutations, 29 were located in exons, including 16 mutations in 3′-untranslated regions (3′-UTR), 4 in 5′-untranslated regions (5′-UTR), and 9 in coding regions (CR) with 4 mutations being synonymous (see Table S2).

**Figure 1:**
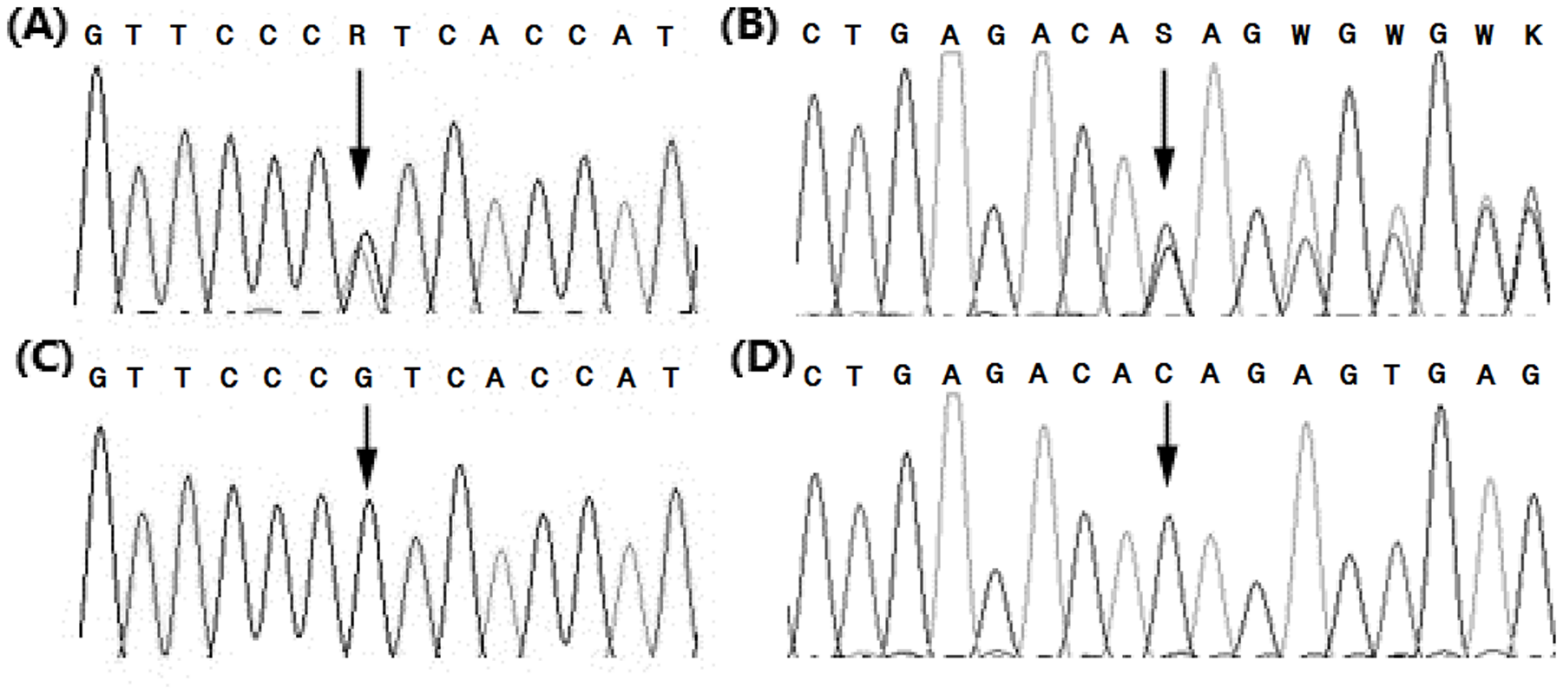
Sequence tracks encompassing c.1303G>A in the bystin-like (*BYSL*) gene and c.1224_1225delCA in the molybdenum cofactor synthesis step 1 (*MOCS1*) gene. (A) and (C) show partial sequences of the *BYSL* gene covering the c.1303G>A mutation. Products were amplified with the DNA of the AS-carrier bull ROMEL and the pooled DNA of three non-carrier bulls, respectively. (B) and (D) are sequencing results covering the c.1224_1225delCA mutation in *MOCS1*. Products were amplified with the DNA of the AS-carrier bull ROMEL and the pooled DNA of three non-carrier bulls, respectively. In (B) we see overlapping sequences after the deletion. The arrows indicate the variant positions.

Supposing that a mutation was the causal site of AS, the presence of the mutation should be in accord with the AS phenotype. In other words, the genotype of the AS-carrier (ROMEL) at this site should be heterozygous (mt/wt) while that of other non-carrier bulls should be homozygous wild-type (wt/wt). Among 48 mutations, there were only 3 mutations supporting the hypothesis (Table 1). The point mutation c.*1975G>A in the 3′-UTR of the *TAF8* gene is not located in any predicated regulatory RNA motifs or elements, indicating a negligible effect at mRNA transcription level; however, the other two mutations, a single base substitution c.1303G>A in *BYSL* gene and a 2-bp deletion c.1224_1225delCA in *MOCS1* gene (Figure 1), are non-synonymous mutations. The variant c.1303G>A was in exon 8 of the *BYSL* gene and would cause a substitution of Val to Ile at the 435^th^ amino acid of the bystin-like protein. However, valine in this position is not highly conserved cross species. For example, the amino acid at this position in human is also isoleucine.

**Table 1:** The three mutations that are in accord with the arachnomelia phenotype.

Gene	Mutation^1^	Position in gene	Position in transcript	Change in amino acid	Genotype (mt, wt)^2^	
					ROMEL^3^	Pooled DNA^4^
*BYSL*	c.1303G>A	exon 8	CR	Val-Ile	mt/wt	wt/wt
*TAF8*	c.*1975G>A	exon 9	3'-UTR	–	mt/wt	wt/wt
*MOCS1*	c.1224_1225delCA	exon11	CR	frame-shift	mt/wt	wt/wt

1 Mutations were recorded according to HGVS (Human Genome Variation Society, http://www.hgvs.org/). The physical position is according to the UCSC genome browser (http://genome.ucsc.edu) based on Bos_taurus_UMD_3.1.

2 mt represents the mutant allele; wt represents the wild-type allele.

3 ROMEL is a known carrier of arachnomelia syndrome.

4 Pooled DNA is the mixture of equal amount of DNA from three known non-carriers of arachnomelia syndrome.

CR: coding region, UTR: untranslated region.

A frame-shift and hence a protein shortened by 176 amino acid (457 aa vs 633 aa) would be generated by the c.1224_1225delCA mutation in exon 11 of the *MOCS1* gene. Structural domain prediction showed that the normal MOCS1a protein translated by *MOCS1* contains 2 structural domains, elongator protein 3 (Elps) and Molybdopterin cofactor biosynthesis C (MoaC). The deletion leads to a possible loss of function of the crucial MoaC domain, which is responsible for biosynthesis of molybdenum cofactor (Moco). Moco is the key component of oxidoreductases in carbon, nitrogen and sulphur metabolism. Therefore, the 2-bp deletion in *MOCS1* is the most likely candidate responsible for AS in Simmental.

### Genotyping in a large population

To further verify this mutation, c.1224_1225delCA in *MOCS1*, the genotype distribution was analyzed in a large cattle population in China. Sample information is shown in Table S3.

**Figure 2:**
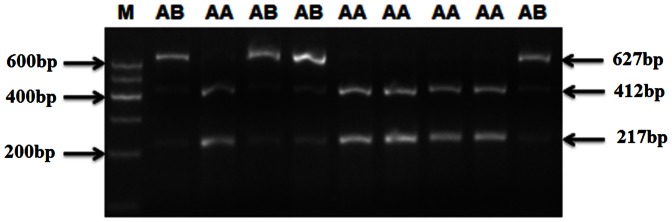
Polymerase chain reaction-restriction fragment length polymorphism analysis of the c.1224_1225delCA mutation. M is 100 bp DNA ladder (Qiagen, Germany); AA represents homozygous normal animals (wt/wt type), with two bands, 412 bp and 217 bp; AB represents AS-carrier animals (mt/wt type), with three bands, 627 bp, 412 bp, and 217 bp.

With the described PCR-RFLP method, genotypes of all 383 samples were tested. As shown in Figure 2,the normal individual (wt/wt), was identified by the presence of two bands, 412 bp and 217 bp; while the AS-carrier (mt/wt), was represented by bands 627 bp, 412 bp, and 217 bp. In our study, no homozygous mutant individual (represented by only one band of 627 bp) was observed.

All 24 progeny of the German Simmental bull ROMEL were parentage tested and confirmed to be the likely offspring. Scanning of the mutation in the 383 samples revealed only 13 individuals, ROMEL and his 12 progeny, carried c.1224_1225delCA in *MOSC1*, indicating the disease-causing mutation segregated with a ratio of 1∶1 in the 24 progeny. The other 370 individuals were homozygous wild-type.

## Discussion

In this study, a network-based disease gene prioritization approach [11] was applied, and genes located within the previously reported ∼7 Mb region on BTA 23 [5] were ranked according to their relevance to AS. By sequencing highly ranked candidate genes, the 2-bp deletion in bovine *MOCS1* gene was successfully identified as the likely disease-causing mutation for AS in Simmental cattle, which confirmed the previous result published by Buitkamp et al. [6]. To our knowledge, this is the first time that the method of combining a network-based candidate gene optimization strategy for scoring and ranking candidate genes and sequencing the most prioritized ones to identify mutations causing simple Mendelian disorders has been successfully implemented.

Typically, for gene discovery the first step is using linkage analysis to anchor a relevant interval at cM-level. Next, traditional fine mapping is used to narrow the region to a small interval and then each of a handful of genes is assessed for their potential functional relevance to the disease and screened for causative mutations [12], [13]. However, this procedure is laborious as well as time-consuming, requiring a large number of individuals with pedigree information to ensure the power and reliability of the results [14], [15]. With the rapid increase of publicly available datasets (e.g., genome draft sequences, biomedical records in life science, and expressing profile), this process can be expedited by prioritizing candidate genes using sophisticated rank aggregation algorithms and data mining prior to testing. Prioritization of candidate genes using a scoring system, first introduced by Perez-Iratxeta et al. [16], is being developed and aimed specifically at finding genes functionally related to complex diseases and quantitative traits based on their association with the biological process of interest. Once a prioritized list is created, the most promising genes can be selected for further analysis. This approach seems to be easier, more economical, and more feasible [17] than previous methods. Network-based candidate gene prioritization was applied to provide new insight into identification of candidate genes underlying diseases or complex quantitative traits. This is favorable to scanning genes at random with low probability of success and may be a complementary approach to QTL mapping.

According to the candidate gene ranking list, *BYSL* gene got the highest z-score value and the reported causative gene *MOSC1* was ranked the sixth. In theory, if there was no prior knowledge, candidates with positive z-score should be analyzed one by one to ensure finding the actually causative mutation. However, in the current study, because the 2-bp deletion in the bovine *MOCS1* gene has been previously reported, genes listed after No. 6 were not evaluated. The causal mutation was detected by investigating top ranked candidate genes in four animals, one known AS-carrier and a pooled sample of three known non-carriers In addition, progeny and dams of the four bulls, as well as unrelated bulls from other breeds/lines were screened to verify the mutation. Among 383 cattle from different breeds/lines, only the AS-carrier bull ROMEL and half of his offspring were heterozygous for the mutation. Our results indicate that c.1224_1225delCA in *MOCS1* causes AS in Simmental and strongly support the conclusion of Buitkamp et al. derived by fine mapping approach [6]. This case indicated that even without large family or population based resources, the network-based disease gene prioritization approach can assist in locating possible causal mutations. Nevertheless, when using a gene prioritization method in a *de novo* mutation screening for complex diseases or traits, the sample size of animals need to be increased for confirmation of results.

Furthermore, an easy genetic detection platform, a PCR-RFLP method was developed for the c.1224_1225delCA mutation in *MOCS1*. With this method, individuals carrying the 2-bp deletion can be quickly identified. Currently, the unfavorable AS allele appears to be not widely existed in the Chinese cattle population. To prevent further spreading of AS among Chinese cattle, all seed-stock animals of Simmental origin, especially AI bulls, should be screened for the causal mutation of AS unless they can be excluded by pedigree. In other words, those progeny born to two negative parents need not be tested.

## Materials and Methods

### Ethics statement

Blood samples from cows were collected by superficial venipuncture of a tail vein using EDTA blood tubes and ear samples from calves were collected while tagging by trained veterinarians. Samples were collected specifically for this study following standard procedures and relevant national guidelines with the full agreement of farmers who owned the animals. Semen samples were contributed by the semen collection centers and were collected following standard semen producing procedures. Formal ethical approval is waived according to the institutional review board of China Agricultural University Animal Ethics Committee.

### Animals and the extraction of DNA

Four German Simmental bulls with acknowledged AS status, including one AS-carrier ROMEL and a pooled sample of three non-carriers (BOSSAG, RIFURT and HIRMER), were used to sequence candidate genes in order to detect possible mutations. The AS disease status of these bulls were determined by pedigree analysis or indirect DNA test [5] and is available online (http://www.zar.at/article/archive/18981). Furthermore, to verify the putative AS causative mutation, samples of 92 cows mated artificially using semen of the four Simmental bulls and their resulted 93 progeny, together with 194 unrelated bulls from 10 cattle breeds/lines were collected. Detailed sample information is included in Table S3.

Genomic DNA of the semen samples was extracted by the traditional phenol-chloroform approach and DNA from ear or blood samples was isolated using the Qiagen DP304 and DP318 commercial kit. Concentrations of genomic DNA were adjusted to 50 ng/µl and the DNA samples were kept at 4°C.

### Candidate genes selection

A network-based disease candidate gene optimization approach, described by Jiang et al.[11], was implemented to rank all coding genes located in the previously reported ∼7 Mb region on BTA 23 limited by the markers *DIK4340* and *BM1815* for their potential roles in AS. Characterization of AS morphology described by Buitkamp et al. [3] was used as disease phenotype. Optimization started from a candidate gene list, including all genes located in the region. Then, a candidate complex was considered based on protein-protein interactions (PPI) from the STRING database (http://string-db.org/). Gene-associated phenotypes were obtained from text mining of biomedical records in the OMIM (http://www.ncbi.nlm.nih.gov/omim), PubMed (http://www.ncbi.nlm.nih.gov/pubmed/), or GeneRIF (ftp://ftp.ncbi.nih.gov/gene/GeneRIF/) databases linked to human, mouse and cattle. The standardized enrichment score (z-score) per gene was computed using a random-set scoring model [18] to illustrate its relative importance to AS. At last, genes were ranked according to the value of z-score.

### Sequencing

To search for possible causative mutations, the top 6 genes out of 27 ranked genes were explored. Basic information regarding each gene is shown in Table 2.

**Table 2:** Information of the six genes which were top ranked by a network-based disease gene prioritization approach.

Rank	Gene	Entrez	Physical position (bp)^1^	Gene length (bp)	Exons length (bp)	Amplification length^2^ (bp)
1	*BYSL*	514128	chr23:16,310,951–16,320,246	9296	2945	5241
2	*TAF8*	539938	chr23:16,418,772–16,438,527	19756	3520	6285
3	*RNF8*	515933	chr23:11,605,616–11,643,036	37421	1988	3620
4	*CDKN1A*	513497	chr23:10,906,547–10,914,830	8248	2352	3634
5	*TBC1D22B*	615878	chr23:11,498,037–11,571,428	73392	2530	6470
6	*MOCS1*	281917	chr23:14,397,224–14,431,707	34484	2678	5593

1 The physical position is according to the UCSC genome browser (http://genome.ucsc.edu) based on Bos_taurus_UMD_3.1.

2 All exons and partial intronic regions were sequenced, so the amplification length was longer than the exons length.

Exons and flanking intronic regions of these 6 prioritized genes were sequenced and compared. Fragments covering coding regions of those genes were amplified from the DNA of the 4 Simmental bulls. Genomic DNAs of the three non-carrier bulls were pooled to reduce costs, and that of the AS-carrier ROMEL was used as the positive control. Primers were designed using Primer3 online (http://primer3.wi.mit.edu/) and Oligo6 primer analysis software (http://www.oligo.net), based on the Bos_taurus_UMD_3.1 reference sequence from the UCSC genome browser (http://genome.ucsc.edu). PCRs were carried out in a total volume of 20 µl, containing 100 ng DNA template, 1 µM of each primer, 200 µM dNTPs, 2.5 unites of Taq DNA polymerase (Takara, Japan), 2 µl of 10×Buffer (Takara, Japan), and 1.5 to 2 mM MgCl_2_. For fragments longer than 900 bp, LA taq polymerase (Takara, Japan) was used. PCR cycling was performed in a PTC-200 DNA Tetrad Thermal Cycler (Bio-Rad, USA) as follows: 94°C for 5 min, followed by 30 cycles of 94°C for 20 s, 56∼65°C for 30 s, and 72°Cfor 30 s, with a final extension completed at 72°C for 7 min. The detail information about primers, annealing temperatures and product characteristics are shown in Table S4.

PCR products were size-separated by electrophoresis on a 2% agarose gel stained with ethidium bromide, extracted from the gel using QiAquick gel extraction kit (Qiagen, Germany) and sequenced from both directions using a ABI BigDye Terminator Sequencing Kit (Applied Biosystems, USA) on an ABI 3730 sequencer. Sequencing peak charts were viewed and compared using Chromas [19]. Sequences of the known AS-carrier and non-carriers as well as the reference sequence from Bos_taurus_UMD_3.1 were multi-aligned with ClustalW in BioEdit software (http://www.mbio.ncsu.edu/bioedit/bioedit.html). Mutation sites were recorded according to the HGVS (http://www.hgvs.org/) recommendation.

### Bioinformatic prediction

Bioinformatic predictions including protein sequence prediction with the ExPASy Proteomics Server (http://www.expasy.org/), cross-species ontology protein multi-alignment with the BioEdit software (http://www.mbio.ncsu.edu/bioedit/bioedit.html), conserve domain prediction with the SMART online software (http://smart.embl.de/), and regulatory RNA motifs identification with RegRNA 2.0 (http://regrna2.mbc.nctu.edu.tw/) were conducted to analyze the suspicious mutations.

### PCR-RFLP

Using the NEB Cutter V2.0 (http://tools.neb.com/NEBcutter2/), we found that the c.1224_1225delCA mutation disrupts a restriction enzyme *DraIII* recognition site and could be identified in a PCR-RFLP test. The primers used were: forward, 5′-ATGAAGGGACAGAGTGGTCGT-3′, and reverse, 5′-CGTGGGTCAGTTGGTCAGAGT -3′.

The PCR was performed under conditions described above. A final volume of 15 µl mixture containing 8 µl of each PCR product, 1.5 µl of NEB buffer 3, 0.15 µl 100×BSA, 0.35 µl restriction enzyme *DraIII* (2 units) and 5 µl double-distilled water was digested at 37°C for 12 h. Subsequently, the mixture was analyzed by 2% agarose gel electrophoresis and accuracy of genotyping results was confirmed by direct sequencing in random samples.

### Parentage testing

Parentage testing was carried out to confirm that the bull ROMEL is the likely sire of his 24 recorded progeny using Bovine Marker Genotyping Kit (Applied Biosystems, USA) according to the manufacturer’s instruction.

## Supporting Information

Table S1 **Candidate genes list ranked by optimization strategy.**
(DOC)

Table S2 **Forty-eight mutations in the top six high-ranking genes derived in this study and their association to Simmental arachnomelia syndrome.**
(DOC)

Table S3 **Information of all samples used in this study.**
(DOC)

Table S4 **Primers used for amplification of the top ranked candidate genes.**
(DOC)
