# Supplementary material for: Identification of the Causative Gene for Simmental Arachnomelia Syndrome Using a Network-Based Disease Gene Prioritization Approach
Source: PLoS One. 2013 May 16;8(5):e64468. doi: 10.1371/journal.pone.0064468 (PMC3655968; doi:10.1371/journal.pone.0064468)
Supplement: Table S1 — Candidate genes list ranked by optimization strategy. (DOC) [file pone.0064468.s001.doc]

**Table S1.** Candidate genes list ranked by optimization strategy

| Rank | Gene abbreviation | Entrez | Z-score | Gene name |
| --- | --- | --- | --- | --- |
| 1 | *BYSL* | 514128 | 10.523 | bystin-like |
| 2 | *TAF8* | 539938 | 7.3928 | ATA box binding protein (TBP)-associated factor |
| 3 | *RNF8* | 515933 | 6.296 | ring finger protein 8 |
| 4 | *CDKN1A* | 513497 | 4.9287 | cyclin-dependent kinase inhibitor 1A |
| 5 | *TBC1D22B* | 281917 | 3.1635 | TBC1 domain family, member 22B |
| 6 | *MOCS1* | 615878 | 2.7579 | molybdenum cofactor synthesis step 1 |
| 7 | *APOBEC2* | 509619 | 2.3665 | apolipoprotein B mRNA editing enzyme, catalytic polypeptide-like 2 |
| 8 | *KCTD20* | 514967 | 2.3138 | potassium channel tetramerisation domain containing 20 |
| 9 | *MED20* | 540294 | 2.0439 | mediator complex subunit 20 |
| 10 | *GL01* | 540335 | 1.8511 | glyoxalase I |
| 11 | *MRPS10* | 515885 | 1.6206 | mitochondrial ribosomal protein S10 |
| 12 | *FRS3* | 509838 | 1.5315 | fibroblast growth factor receptor substrate 3 |
| 13 | *NFYA* | 539584 | 1.4857 | nuclear transcription factor Y, alpha |
| 14 | *PI16* | 507058 | 1.3356 | peptidase inhibitor 16 |
| 15 | *PNPLA1* | 786888 | 1.3356 | patatin-like phospholipase domain containing 1 |
| 16 | *TREM2* | 506467 | 1.1529 | triggering receptor expressed on myeloid cells 2 |
| 17 | *MAPK13* | 535327 | 0.9954 | mitogen-activated protein kinase 13 |
| 18 | *TOMM6* | 505031 | 0.8017 | translocase of outer mitochondrial membrane 6 homolog (yeast) |
| 19 | *KCNK17* | 282264 | 0.7478 | potassium channel, subfamily K, member 17 |
| 20 | *PIM1* | 281402 | 0.5918 | pim-1 oncogene |
| 21 | *PPIL1* | 508179 | 0.1996 | peptidylprolyl isomerase (cyclophilin)-like 1 |
| 22 | *BTBD9* | 505504 | -0.8087 | BTB (POZ) domain containing 9 |
| 23 | *CCND3* | 540547 | -2.9283 | cyclin D3 |
| 24 | *GUCA1B* | 286851 | -3.0484 | guanylate cyclase activator 1B (retina) |
| 25 | *TREM1* | 404547 | -3.6472 | triggering receptor expressed on myeloid cells 1 |
| 26 | *GUCA1A* | 282243 | -4.2044 | guanylate cyclase activator 1A (retina) |
| 27 | *MAPK14* | 534492 | -14.8149 | mitogen-activated protein kinase 14 |

The candidate genes were ranked according to the standardized enrichment score (z-score). Z value was calculated according to Jiang et al. (2012). A large positive z-score favors that the candidate gene is strongly associated with Simmental arachnomelia syndrome.
