# Supplementary material for: Identification of the Causative Gene for Simmental Arachnomelia Syndrome Using a Network-Based Disease Gene Prioritization Approach
Source: PLoS One. 2013 May 16;8(5):e64468. doi: 10.1371/journal.pone.0064468 (PMC3655968; doi:10.1371/journal.pone.0064468)
Supplement: Table S2 — Forty-eight mutations in the top six high-ranking genes derived in this study and their association to Simmental arachnomelia syndrome. (DOC) [file pone.0064468.s002.doc]

**Table S2.** Forty-eight mutations in the top six high-ranking genes derived in this study and their association to Simmental arachnomelia syndrome

| Candidate gene | Mutation^1^ | Position in gene | Position in transcript | Amino acid change | Genotype (mt, wt) ^2^ | |
| --- | --- | --- | --- | --- | --- | --- |
|  |  |  |  |  | ROMEL^3^ | Pooled DNA^4^ |
| ***BYSL*** | | | | | | |
|  | c.-248G>A | exon 2 | 5’-UTR | N | mt/mt | mt/wt |
|  | c.-256A>T | exon 2 | 5’-UTR | N | wt/wt | mt/wt |
|  | c.565-8C>G | intron 4 | - | - | mt/wt | mt/mt |
|  | c.1303G>A | exon 8 | CR | Val-Ile | mt/wt | wt/wt |
|  | c.*29G>T | exon 8 | 3’-UTR | N | mt/wt | mt/mt |
|  | c.*154G>A | exon 8 | 3’-UTR | N | mt/mt | mt/mt |
|  | c.*758C>T | exon 8 | 3’-UTR | N | mt/wt | mt/mt |
|  | c.*1069G>A | exon 8 | 3’-UTR | N | mt/wt | mt/mt |
|  | c.*1627+62C>T | intron 9 | - | - | mt/wt | mt/mt |
| ***TAF8*** | | | | | | |
|  | c.*838T>C | exon 9 | 3’-UTR | N | mt/wt | mt/wt |
|  | c.*1620C>T | exon 9 | 3’-UTR | N | mt/wt | mt/wt |
|  | c.*1700C>G | exon 9 | 3’-UTR | N | mt/wt | mt/wt |
|  | c.*1941C>G | exon 9 | 3’-UTR | N | mt/wt | mt/wt |
|  | c.*1975G>A | exon 9 | 3’-UTR | N | mt/wt | wt/wt |
|  | c.*3039A>G | exon 9 | 3’-UTR | N | mt/wt | mt/wt |
|  | c.*2208A>G | exon 9 | 3’-UTR | N | mt/wt | mt/wt |
| ***RNF8*** | | | | | | |
|  | c.240+49A>T | intron 2 | - | - | mt/mt | mt/wt |
|  | c.1545-183delA(13_15) | intron 4 | - | - | mt/wt | mt/wt |
|  | c.*41A>G | exon 8 | 3’-UTR | N | mt/mt | mt/mt |
|  | c.*310_311insATTT | exon 8 | 3’-UTR | N | mt/mt | mt/mt |
| ***CDKN1A*** | | | | | | |
|  | c.-7+89A>G | intron 1 | - | - | mt/wt | mt/mt |
|  | c.-7+248C>T | intron 1 | - | - | wt/wt | mt/wt |
|  | c.271C>T | exon 2 | CR | Arg-Trp | wt/wt | mt/wt |
|  | c.437-100T>A | intron 2 | - | - | mt/wt | wt/wt |
|  | c.437-86T>A | intron 2 | - | - | mt/wt | wt/wt |
| ***TBC1D22B*** | | | | | | |
|  | c.-93G>T | exon 1 | 5’-UTR | N | mt/wt | mt/wt |
|  | c.57-9G>A | intron 1 | - | - | mt/wt | wt/wt |
|  | c.478T>G | exon 3 | CR | Ile-Ser | mt/mt | mt/mt |
|  | c.596C>T | exon 4 | CR | synonymous | wt/wt | mt/mt |
|  | c.709-41C>T | intron 4 | - | - | mt/mt | mt/wt |
|  | c.779+42A>G | intron 5 | - | - | mt/mt | mt/mt |
|  | c.779+50C>A | intron 5 | - | - | mt/mt | mt/mt |
|  | c.1161C>T | exon10 | CR | synonymous | mt/mt | mt/wt |
|  | c.1165+42A>G | intron 10 | - | - | mt/mt | mt/wt |
|  | c.1294-106T>C | intron 11 | - | - | mt/mt | mt/wt |
|  | c.1497-51G>A | intron 12 | - | - | wt/wt | mt/wt |
|  | c.1497-39G>T | intron 12 | - | - | wt/wt | mt/wt |
|  | c.1494C>T | exon 13 | CR | synonymous | wt/wt | mt/wt |
|  | c.*308T>G | exon 13 | 3’-UTR | N | wt/wt | mt/wt |
|  | c.*389_390insTA | exon 13 | 3’-UTR | N | mt/wt | mt/mt |
|  | c.*829T>C | exon 13 | 3’-UTR | N | mt/mt | mt/wt |
| ***MOCS1*** | | | | | | |
|  | c.-1A>G | exon1 | 5’-UTR | N | wt/wt | mt/wt |
|  | c.399+5G>T | intron3 | - | - | wt/wt | mt/wt |
|  | c.711C>T | exon6 | CR | synonymous | mt/mt | mt/wt |
|  | c.898-12G>A | intron6 | - | - | wt/wt | mt/wt |
|  | c.1152G>A | exon10 | CR | Met-Ile | mt/wt | mt/wt |
|  | c.*10-12 | intron10 | - | - | mt/wt | mt/wt |
|  | c.1224_1225delCA | exon11 | CR | frame-shift | mt/wt | wt/wt |

^1^ Mutations were recorded according to HGVS (Human Genome Variation Society, http://www.hgvs.org/). The physical position is according to the UCSC genome browser (http://genome.ucsc.edu) based on Bos_taurus_UMD_3.1.

^2^ mt represents the mutated allele; wt represents the wild allele.

^3^ ROMEL is a known carrier of arachnomelia syndrome.

^4^ Pooled DNA is the mixture of equal amount of DNA from three known non-carriers of arachnomelia syndrome.

CR: coding region, UTR: untranslated region.
