# Supplementary material for: Identification of the Causative Gene for Simmental Arachnomelia Syndrome Using a Network-Based Disease Gene Prioritization Approach
Source: PLoS One. 2013 May 16;8(5):e64468. doi: 10.1371/journal.pone.0064468 (PMC3655968; doi:10.1371/journal.pone.0064468)
Supplement: Table S3 — Information of all samples used in this study. (DOC) [file pone.0064468.s003.doc]

**Table S3. Information of all samples used in this study**

| Animal | Breed | No. | Source |
| --- | --- | --- | --- |
| bull | German Simmental | 4 | Besamungsverein Neustadt a.d. Aisch e.V. (BVN, Germany) |
|  | Australian Simmental | 28 | Beijing Dairy Cattle Center, Inner Mongolia livestock improvement station, Henan Dingyuan Seedstock Bulls Breeding Ltd. Co |
|  | Chinese Simmental | 7 | Xinjiang General Livestock Service |
|  | Montbeliarde | 12 | Coopex, French |
|  | Angus | 9 | Beijing Dairy Cattle Center, Henan Dingyuan Seedstock Bulls Breeding Ltd. Co, Ningxia Sygen BioEngineering Research Center |
|  | Wagyu | 11 | Beijing Dairy Cattle Center, Dalian Xuelong Industry Limited Group, Dalian |
|  | Holstein | 19 | Beijing Dairy Cattle Center, Ningxia Sygen BioEngineering Research Center |
|  | Brown Swiss | 59 | Beijing Dairy Cattle Center, Xinjiang Tianshan Animal Husbandry Bio-engineering Co. Ltd, Xinjiang General Livestock Service |
|  | Limousin | 15 | Beijing Dairy Cattle Center, Henan Dingyuan Seedstock Bulls Breeding Ltd. Co, Luoyang bull station, Henan |
|  | Norwegain Red | 4 | Geno breeding & AI association |
|  | Charolais | 30 | Beijing Dairy Cattle Center, Henan Dingyuan Seedstock Bulls Breeding Ltd. Co, Luoyang bull station, Xuchang bull station, Henan |
| dam | Holstein | 82 | Anshan Hengli Dairy Farm, Liaoning |
|  | Sanhe | 10 | Xieertala Breeding Farm, Inner Mongolia |
| calf | German Simmental×Holstein | 80 | Anshan Hengli Dairy Farm, Liaoning |
|  | German Simmental×Sanhe | 13 | Xieertala Breeding Farm, Inner Mongolia |
| Total |  | 383 |  |
