# Supplementary material for: Identification of the Causative Gene for Simmental Arachnomelia Syndrome Using a Network-Based Disease Gene Prioritization Approach
Source: PLoS One. 2013 May 16;8(5):e64468. doi: 10.1371/journal.pone.0064468 (PMC3655968; doi:10.1371/journal.pone.0064468)
Supplement: Table S4 — Primers used for amplification of the top ranked candidate genes. (DOC) [file pone.0064468.s004.doc]

**Table S4.** Primers used for amplification of the top ranked candidate genes

| Candidate gene | Primer name | Forward primer | Reverse primer | Start (bp) | End (bp) | Product length(bp) | Annealing temperature(℃) |
| --- | --- | --- | --- | --- | --- | --- | --- |
| *BYSL* | BY-1 | CCACACAAACCGAGGACATT | AGGACCTAGACACGCCCTTC | 15696511 | 15696840 | 330 | 61 |
|  | BY-2 | TGGAAGGATCAAACAACTGAAA | TACCCACTCTCCTCCTCGTACC | 15696681 | 15697501 | 821 | 56 |
|  | BY-3 | ATGGTTAGAGTTGAGTTGACCT | GTTTCTTTCCTACGCAGTTGCT | 15701290 | 15701589 | 300 | 65 |
|  | BY-4 | GATTCTTTGCTGGGTGGGTAG | TTGAGATTAGAGTCATAGCGGG | 15703424 | 15703837 | 414 | 58 |
|  | BY-5 | GATGGTTGTGAGGATTGAGTGAG | AGCCTGCCCTATTGACCCAA | 15703881 | 15704156 | 276 | 58 |
|  | BY-6 | TCATCGGCAGTGGTATTTGAA | GGCTTATGCTTGACTCCTTCC | 15704611 | 15705163 | 553 | 63.5 |
|  | BY-7 | CCTTTAGGGACAGCACAATTTA | TTGAGAACTGGGGAGTCAGAG | 15705449 | 15706029 | 581 | 56 |
|  | BY-8 | GCTGGTGACTGGAGGTGATT | GGTGCTTTCTTGTAGCTCTTCC | 15705938 | 15706517 | 580 | 61 |
|  | BY-9 | CACCTTATTCTACAAGTCTTCT | GTTGGAACAAACTCATATTC | 15706349 | 15706916 | 568 | 56 |
|  | BY-10 | GACTGCTTTCACTGCTCCTG | AATGGGGCTTGAGACAATACTC | 15706829 | 15707283 | 455 | 55 |
|  | BY-11 | TGATTTCTGCCTTTTACCCC | CTGTATTCCTCCCCTCTGCTAC | 15707469 | 15707831 | 363 | 61 |
| *TAF8* | TAF8-1 | CTTAACTACAAGTCCCGTCGT | GAAAGACCCTCCTCCACGTA | 15803510 | 15803742 | 233 | 58 |
|  | TAF8-2 | GCCGCTTTGTGTTATGGAAT | CAGATGTTTGACTGGAAGAAGG | 15804161 | 15804603 | 443 | 58 |
|  | TAF8-3 | GAGTGGAGTTGCTGGCTCATA | ACGAGGAGATTCAAGGACATTC | 15807261 | 15807560 | 300 | 63.5 |
|  | TAF8-4 | CAGCATTACTTCCCCTTCCAT | ATAACCAGTCCCTGCCCTATTC | 15807705 | 15807916 | 212 | 58 |
|  | TAF8-5 | CCAGCGTTTATTTTGAGAAA | TTTGGAGGAGAGACATTCTGA | 15808716 | 15809057 | 342 | 56 |
|  | TAF8-6 | AGGACAGAGCAACAGCAAGG | ACAATGAGGGAGGGAAGAGAG | 15812078 | 15812377 | 300 | 65 |
|  | TAF8-7 | TAGTAAGGTGGAGATGAGGATT | CATTCAGTATCTGCTGGATGAC | 15813526 | 15814010 | 485 | 58 |
|  | TAF8-8 | ACTCCCGCCTTCCTCTGAT | GAACACTTCCTGTCACCCAAG | 15820032 | 15820482 | 451 | 61 |
|  | TAF8-9 | AGCAGTTTTTGTTCCCCTCAC | ACTAAGCACACCGTCTTTCTGG | 15820579 | 15821028 | 450 | 58 |
|  | TAF8-10 | GGAGACGAGAGAAGGTAGCAGA | ATCAAGGATGAAGAGGCAAAGA | 15820953 | 15821695 | 743 | 56 |
|  | TAF8-11 | GTCCAAAGGCTGAGAAACCTG | ACAGACGGGGGAAACTCTTATT | 15821556 | 15822114 | 559 | 61 |
|  | TAF8-12 | TCTCTAAACACATCATCCCACTG | CTACTTTTTCCCTCCACGCAT | 15821992 | 15822542 | 551 | 58 |
|  | TAF8-13 | GCTGTAATGCTGAGTGATGAG | GCAGACTTCCCAAATAAAAAA | 15822312 | 15822997 | 686 | 56 |
|  | TAF8-14 | GCGACTTAGCAGCAATAAAAGG | ACACACACACACACAAAGGTGA | 15822836 | 15823365 | 530 | 65 |
| *RNF8* | RNF8-1 | CAAGCGAACCTTCACAACGAT | ACTTCTCCCAACTCCTCCTTCC | 11229703 | 11230075 | 373 | 63.5 |
|  | RNF8-2 | AATACTGCTGGTTGACAAGAC | ATACCTATGATTGATTCACATTG | 11239845 | 11240191 | 347 | 55 |
|  | RNF8-3 | CAGCAGCAGGAGAAAAATTAAGA | TCTTCACACTTGGCTCAAAAGA | 11250345 | 11250820 | 476 | 56 |
|  | RNF8-4 | CACATGGGAAGGGTAAAGTGG | CTCTGGTCACAGGCTCACAA | 11250740 | 11251225 | 486 | 58 |
|  | RNF8-5 | ATTTCCCACTCCCTCCCTTC | CCCCCACTCTCACCCACTAT | 11253335 | 11253575 | 241 | 56 |
|  | RNF8-6 | TCCCTCCCTATCTGCTCCTT | CATACCTCTCCCTTCATTCACC | 11256220 | 11256619 | 400 | 58 |
|  | RNF8-7 | GTGTTACTACGCCAGGGTGAAT | CAGGGATGGGAGAGTGAGAAG | 11258165 | 11258451 | 287 | 58 |
|  | RNF8-8 | CTTGAGAACTAACTCCTTTCGT | TAACTCTGTTGAGGGGACTC | 11260518 | 11260860 | 343 | 58 |
|  | RNF8-9 | AAGAGAAGGGTGGGCTGTAAG | GCTGGAAGGAAAGGAAGATAAA | 11266537 | 11267203 | 667 | 56 |
| *CDKN1A* | CDK-1 | GGAGAGTGGGAAGGAGGGAAGC | TCCAGGGGAGGGATCGCAGA | 10560343 | 10560886 | 544 | 58 |
|  | CDK-2 | CTTTCTAGTAAGGAGACTGCG | TGAGTTCTGGGAGTCCTAATC | 10564943 | 10565554 | 612 | 58 |
|  | CDK-3 | ATGTTCTGTCTGTATGTAGCCC | CTCTGAGCAAAGGATGAAGAG | 10566796 | 10567313 | 518 | 58 |
|  | CDK-4 | ACATACCCTGCTTGCTGCCACC | TTTGATGACGCCTCCAACTCTG | 10567155 | 10567808 | 654 | 58 |
|  | CDK-5 | GCCATCTGCTGGGTGTTACGAA | GTGTCACACTGTCCCTCCTGGA | 10567680 | 10568375 | 696 | 58 |
|  | CDK-6 | AGTTCTACCTCAAGCAGCGACT | AAACCTCCACCTACTTGCTCTT | 10568230 | 10568839 | 610 | 61 |
| *TBC1D22B* | TB-1 | GGAGTGGTGGGAAGGATAATAA | CGGGATTTAGGGTTTAGGATTT | 11132581 | 11133267 | 688 | 61 |
|  | TB-2 | AAACCTCACCAGGGAAGATGAT | GGTCAAAGCAGAAACTGTCACT | 11148257 | 11148600 | 344 | 58 |
|  | TB-3 | TTATTTGAAGAGGGTAATGATTG | CAGGGAAAGGAAGGAGAGAA | 11159351 | 11160039 | 689 | 58 |
|  | TB-4 | CAGCAGTCAGGTCTAAGGTGT | TGGAAAAGTAAAAGAAGGAAGAA | 11161943 | 11162317 | 375 | 55 |
|  | TB-5 | CCTCTAACTCTAATCTCTGAGC | GACTATCCACACAAACCTAAGC | 11162512 | 11163027 | 516 | 58 |
|  | TB-6 | GCTTTCTTTCCCTTCCTTTGAA | TTCCTAACCACTGTAGCCTGAA | 11164074 | 11164427 | 354 | 55 |
|  | TB-7 | GTTTGTTCAGGGAGGTTTCATT | CACTCACCCACACGGACAGT | 11165690 | 11166051 | 362 | 63.5 |
|  | TB-8 | GCATTGGTGGATGGATTCTT | GCACTCTTGGGAAAAACAAA | 11170516 | 11170925 | 410 | 58 |
|  | TB-9 | TAAGGACCCCGTTGAAGATACT | GAGTTCGGAGGCTGTCTGAT | 11188953 | 11189238 | 286 | 56 |
|  | TB-10 | GAGATTCCTTTGTTCCCCACA | CCACCCCACACCAGCATAAT | 11189598 | 11189901 | 304 | 61 |
|  | TB-11 | CCTTTTTGGTTTATGTGAGTCA | GGCAGACACAGAAGCAGAAA | 11192254 | 11192575 | 322 | 56 |
|  | TB-12 | TGTGTCTGCCTTGTCCTCAA | GATAATGCCCACTCTGATGCTC | 11192566 | 11192881 | 316 | 58 |
|  | TB-13 | AAAGAGGCAGGTGTTAGTGGAT | TGTAGGGCAATGTGAGTTGAGA | 11205272 | 11205945 | 674 | 63.5 |
|  | TB-14 | GGAGAGGACAGAAAAGGAGGTA | GGGGTTCGGAGAGGTGATAAG | 11205847 | 11206432 | 586 | 63.5 |
|  | TB-15 | ATACACGCATCCCTTGAGAA | CTGGCAACTTCATCTGTGCTA | 11206365 | 11206608 | 244 | 55 |
| *MOCS1* | MO-1 | CACGTCGATTGGCTGCTC | AGGAAAAGTGTGGATGCGAAC | 13866772 | 13867049 | 278 | 60 |
|  | MO-2 | TATGGATGGAGAGGCAGTGTGT | TAGAAAGAGGAATGGGGAGGAG | 13858199 | 13858560 | 362 | 60 |
|  | MO-3 | GGTGAGTTGGGGACTGGAG | CTGGGAGAGGCGGTGATAA | 13856633 | 13856912 | 280 | 60 |
|  | MO-4 | AGAGCCCGTTTTCTATCCATCT | ACCGTGAACCCCATCTGA | 13843044 | 13843332 | 289 | 60 |
|  | MO-5 | CCCTGGACTCTGTGGTCTCT | CTACACGCTCAGTGGGGTCT | 13840069 | 13840828 | 760 | 56 |
|  | MO-6 | TGACCCAGAGGCAGAGAACT | CCACCACTTACTCCAATGTGC | 13839493 | 13839942 | 450 | 60 |
|  | MO-7 | TCAGGGTTTCTGCTGTTTGAG | ATTTCACCATCACTCCCGTGTA | 13839023 | 13839383 | 361 | 60 |
|  | MO-8 | CCTTACAGCCCCTCTTGACTAC | CCCCCAAACTACGGACCT | 13836576 | 13836957 | 382 | 58 |
|  | MO-9 | GTGCTCTGGGCTCTGTCGT | GCAGGAAAAGATGAAGGATGAG | 13835954 | 13836328 | 375 | 60 |
|  | MO-10 | ATGAAGGGACAGAGTGGTCGT | CGTGGGTCAGTTGGTCAGAGT | 13833649 | 13834277 | 629 | 60 |
|  | MO-11 | GAGTGAGTTTCTCCAGCCAGAT | ACTTGACCTCTTTCCCTTGG | 13833115 | 13833867 | 753 | 56 |
|  | MO-12 | CGTCAGCAGGGACATCGT | GTGAAGGGATACAGGAGGTGTG | 13832553 | 13833272 | 720 | 64 |
|  | MO-13 | ATCAGACACACAGGCTTCCAG | CGACAGGAGGTGCTTTGG | 13832228 | 13832688 | 461 | 58 |

Note: The location of primers was based on Bos_taurus_UMD_3.1 from UCSC genome browser (http://genome.ucsc.edu).
